# Improvement in Upper Limb and Systemic Myalgic Encephalomyelitis/Chronic Fatigue Syndrome (ME/CFS) Symptoms After Surgical Treatment of Neurogenic Thoracic Outlet Syndrome

**DOI:** 10.7759/cureus.90494

**Published:** 2025-08-19

**Authors:** Maritsa E Christoforou, Ying Wei Lum, Sally C Sroge, Alba M Azola, Peter C Rowe

**Affiliations:** 1 Division of Adolescent and Young Adult Medicine, Department of Pediatrics, Johns Hopkins University School of Medicine, Baltimore, USA; 2 Division of Vascular Surgery and Endovascular Therapy, Department of Surgery, Johns Hopkins University School of Medicine, Baltimore, USA; 3 Department of Physical Therapy, Messiah University, Mechanicsburg, USA

**Keywords:** hypermobile ehlers-danlos syndrome, myalgic encephalomyelitis/chronic fatigue syndrome (cfs), neurogenic thoracic outlet syndrome, postural tachycardia syndrome, reversal of vision metamorphopsia

## Abstract

Thoracic outlet syndrome (TOS) is characterized by compression of nerves or blood vessels as they pass through the scalene triangle and the costoclavicular space, and under the pectoralis minor. Common symptoms include arm fatigue and heaviness, paresthesias, and neck and upper back pain, provoked by arm extension or elevation. We have recently reported that some myalgic encephalomyelitis/chronic fatigue syndrome (ME/CFS) patients report symptoms suggestive of TOS, specifically with respect to overhead activity, but there is uncertainty whether this overlap in symptoms is more related to ME/CFS itself or a direct contribution by TOS. This case report describes an ME/CFS patient diagnosed with TOS, who experienced major decreases in many expected and unexpected symptoms after bilateral TOS surgery.

A 19-year-old female patient with ME/CFS and the hypermobile Ehlers-Danlos syndrome (hEDS) developed progressive symptoms of numbness and tingling in the upper limbs, which did not improve after two months of physical therapy. The patient elected to undergo the rib resection with neurolysis and scalenectomy surgery on her left side. Due to the success in the reduction of symptoms, she elected to undergo the same procedure on the right side three months later.

By eight weeks after the second surgery, the patient had experienced an expected complete resolution of upper limb numbness and tingling. She also reported a complete resolution of migraines, occipital neuralgia, vertigo, and visual disturbances, along with a marked improvement in cognitive fogginess and lightheadedness.

This case report highlights the potential for marked improvements in clinical function after recognition and surgical treatment of TOS in a patient with comorbid hEDS and ME/CFS. In addition to expected improvement in upper limb symptoms and the resolution of occipital headaches, our patient noted improvement in systemic symptoms of lightheadedness, cognitive dysfunction, and visual disturbances. This experience suggests that those with hEDS and ME/CFS should be more carefully screened for brachial plexus dysfunction. Conversely, ascertainment of systemic symptoms may enhance the diagnosis of TOS and the items assessed in surgical treatment outcome studies.

## Introduction

Thoracic outlet syndrome (TOS) is caused by a compression of the nerves, veins, and arteries that travel through the thoracic outlet, a region that extends from the lateral neck through the scalene muscles, under the clavicle, over the first rib, and into the anterior chest [1-5]. Descriptions of TOS symptoms usually focus on upper limb pain, fatigue, numbness, and tingling provoked by arm elevation or extension. The most common form of TOS is neurogenic (nTOS), which involves compression of the brachial plexus nerves [6-8].

Myalgic encephalomyelitis/chronic fatigue syndrome (ME/CFS) is a multisystem disorder defined by significant impairment in function, usually presenting with profound fatigue, along with postexertional malaise (PEM), unrefreshing sleep, orthostatic intolerance, and/or cognitive dysfunction [9]. We recently reported that in individuals with ME/CFS, a TOS examination maneuver provoked not only the expected upper limb symptoms but also a number of systemic symptoms such as lightheadedness, fatigue, racing heart, and cognitive fogginess. It was unclear whether the provocation of symptoms was a direct result of brachial plexus dysfunction or a manifestation of PEM, one of the cardinal symptoms of ME/CFS [10].

This case study describes the response of a patient with ME/CFS to bilateral nTOS decompression. She reported substantial improvements in both upper limb symptoms and complete alleviation of migraines, occipital neuralgia, visual disturbances, and vertigo. These improvements lend support to the hypothesis that nTOS is another contributor to symptoms in some patients with ME/CFS. Our findings provide further support for more careful screening of ME/CFS patients for TOS and for more careful ascertainment of a broader range of neurological symptoms when measuring outcomes of TOS treatment.

Questionnaires

Surgical outcomes were assessed using three questionnaires. To evaluate TOS-specific symptoms, the patient completed the shortened version of the Disability of the Arm, Shoulder, and Hand measurement (QuickDASH) survey, an Open Access survey owned by the Institute for Work and Health. The QuickDASH was completed a month before surgery and a month after the second operation [11]. This 11-item survey asks patients to rank the difficulty experienced with common daily tasks involving the upper limbs, interference with social and work activities, and the severity of upper limb pain, numbness, and tingling. The individual responses range from one to five, where one represents no difficulty and five represents unable/extreme disability. Total scores range from 0 to 100, which is calculated based on the following formula, where n is the number of responses [11]:

$\left( \frac{\text{sum of } n \text{ responses}}{n} - 1 \right) \times 25$.

To evaluate overall well-being, we administered the Pediatric Quality of Life Inventory (PedsQL), specifically the version 4.0 Young Adult inventory (Children’s Hospital and Health Center, San Diego, CA), which is validated for ages 18-25 [12]. To evaluate cognitive fatigue, we administered the Wood Mental Fatigue Inventory (WMFI) [13]. Both were completed at the initial ME/CFS evaluation nine months before surgery and two months after the second operation. The PedsQL questionnaire has 23 total items divided into four scales (physical, emotional, social, and school functioning). The individual responds on a scale from zero (never experiences) to four (almost always experiences), and the scale score is then calculated using a reverse scoring system. In the reverse scoring system, a zero is scored as 100, and each subsequent number decreases by 25. The reverse score is then averaged. Scores range from 0 to 100, with a score of 100 meaning perfect mental and physical well-being [12]. Permission to use this questionnaire was granted by Mapi Research Trust, James W. Varni, copyright holder. The WMFI is an open-access nine-item questionnaire scored from 0 to 36, with 0 indicating high cognitive function and low mental fatigue [13].

Surgical intervention

For this individual’s nTOS surgery, a transaxillary approach was used. For this procedure, patients are positioned in the lateral decubitus position. An incision is made between the pectoralis major and the latissimus dorsi in the axilla. Skin and soft tissue are dissected until the chest wall is encountered above the serratus fascia. The borders of the first rib are bluntly dissected using the periosteal elevator. The subclavius muscle and the anterior scalene muscle are divided. This is followed by circumferential dissection of the first rib, freeing the middle scalene muscle from the rib. The bone cutter is used to transect the rib medially and posteriorly, removing as much bone as possible. Next, scissors are used to dissect between the subclavian artery and the trunks of the brachial plexus, thereby identifying the roots of the brachial plexus. The scalene muscle fibers that interdigitate the trunks of the brachial plexus are also removed, as is fibroconnective tissue and scalene muscle remnants in between the T1 and C8 roots. Any remaining scalene muscle fiber or scar tissue surrounding the subclavian artery and vein is removed. After 24 hours of observation in the hospital, patients are discharged with instructions to complete 8-12 weeks of structured physical therapy at an interval of three times a week. For bilateral treatment, the surgery on the opposite side is performed after at least three months of recovery.

Physical therapy testing

All of the following physical therapy tests were completed two months before the first surgery, and one month after each surgery. The Adson’s test is performed by having the patient rotate the neck toward the tested side, while the clinician palpates the radial pulse. A positive result occurs when the radial pulse diminishes or obliterates [5,8]. The elevated arm stress test (EAST) is performed by having the patient sit while holding their arms at a 90° angle above shoulder level. The patient is asked to open and close their fists while the clinician sets a timer for three minutes. A positive result occurs when TOS symptoms of numbness or tingling appear, and the result is accompanied by the time duration until onset in each limb. The levator scapula length test (LSLT) is performed by having the patient raise one arm 180° upward and then repeating this maneuver after the neck is side-bent contralaterally. If the patient cannot bring the arm back to 180°, this is considered a positive result. Cervical range of motion tests were recorded using a goniometer to measure the degree of motion [5,8]. Grip strength was tested using a dynamometer. Three trials were performed in succession on each side and averaged.

## Case presentation

The patient was evaluated at the Johns Hopkins Children’s Center ME/CFS clinic at 19 years of age. In childhood, she had been diagnosed with eczema, IgE-mediated allergies to peanut, avocado, and sunflower, and oral allergy syndrome. Eosinophilic esophagitis (EoE) was recognized at the age of 16 and gastroparesis at the age of 18. The diagnosis of hereditary alpha tryptasemia was confirmed using a TPSAB1 copy number analysis genetic test at the age of 18.

She had symptoms of ME/CFS from age 14 onward, characterized by substantial impairment in activities that were well tolerated before the onset of illness and constant fatigue. She was able to attend school but was unable to participate in extracurricular activities. She would develop PEM after increased physical or cognitive activity, or after orthostatic stress, such as going to the shopping mall. She reported new problems with concentration, short-term memory, and word finding, describing her head as feeling foggy and her thoughts as slow. She slept 10-12 hours per night, awakening unrefreshed, and napping throughout the day. Passive standing tests conducted at home confirmed a >40 bpm increase in heart rate during the first 10 minutes of standing, compared to supine values. The test provoked typical orthostatic symptoms and no evidence of orthostatic hypotension, consistent with postural tachycardia syndrome (POTS) [14].

Headaches began in the summer of 2023, affecting the entire head and behind the eyes, accompanied by occipital migraines with photophobia. Washing her hair and shaving her legs were associated with lightheadedness. She avoided reaching up because this was associated with developing heart pounding, tingling of the upper limbs, and tunneling of her vision.

She was flexible as a child and had a positive family history on the maternal side for joint hypermobility (JH). Physical examination was notable for a Beighton score of 5/9 for apposition of each thumb to the flexor aspect of the forearm, 20° of elbow hyperextensibility bilaterally, the ability to place the palms on the floor with the legs straight, piezogenic papules of the heels, and hyperextensible upper eyelids [15]. Deep tendon reflexes were 2+ and symmetrical except 2-3+ at the patella on the right, and 3+ at the patella on the left, with 5 cm of spread above the patella on the left side.

On palpation, she had very tight trapezius muscles and tender right serratus anterior muscles. A three-minute EAST provoked heart pounding immediately (at five seconds), arm fatigue at 58 seconds, shoulder and upper arm soreness at 70 seconds, increased general fatigue at 80 seconds, finger tingling at 110 seconds, and lightheadedness at 140 seconds. An upper limb tension test with a median nerve bias was normal at the initial evaluation. Six months later, she developed an increase in TOS symptoms, with progressive difficulty with grip strength, dropping things, occipital and upper limb neuralgia, vertigo, and more frequent numbness and tingling. She also began noticing brief reversal-of-vision metamorphopsia (RVM), a 180° inversion of the visual field, accompanying her worst episodes of vertigo and occipital headaches. At this point, the upper limb tension test was now positive (eliciting symptoms upon cervical motion). The EAST was repeated, this time provoking arm fatigue and tingling after only seven seconds. Other examination findings by the physical therapist (S.C.S.) included decreased cervical side bend and rotational range of motion, hypertonicity of the scalenes, hypomobility of the first rib bilaterally, symptom reproduction with median and ulnar nerve gliding, reproduction of symptoms with Adson's and EAST, stiffness of elevator scapular muscles, and weak grip strength.

Despite two months of manual physical therapy, all of the symptoms discussed above were progressing, with constant upper limb paresthesias, worse grip strength, and increased frequency and severity of occipital migraines. Continuation of physical therapy was associated with an immediate increase in symptoms, reflecting the severity of compression of structures in the thoracic outlet, so she was seen in Vascular Surgery by Y.W.L. A Doppler ultrasound showed a right subclavian artery adduction to abduction velocity of 82-98 cm/second and a left subclavian artery adduction to abduction velocity of 81-190 cm/second. The normal range for abduction velocities is between 50 and 100 cm/second, which would categorize the patient’s left side as TOS and the right as normal. The radial pulse was obliterated bilaterally with upper limb tension testing.

Given the worst Doppler findings on that side, the patient underwent left first rib resection with neurolysis and scalenectomy. Intraoperative visualization confirmed extrinsic compression of the left subclavian artery. The patient reported prompt relief following surgery. At the one-month follow-up, the patient reported an estimated 50% improvement in TOS symptoms, primarily defined by the near absence of neuralgia, numbness, or tingling in the left upper limb. At the two-month mark, the patient reported that other symptoms she previously attributed to POTS were improved; this included reduced migraines (from three to one per week) and improved ipsilateral occipital neuralgia, vertigo, and brain fog. Due to the degree of improvement and the contemporaneous increased severity of right TOS symptoms, the patient elected to have the right TOS decompressed three months after the first surgery.

One month after the right-sided surgery, the patient noted that her occipital neuralgia, arm neuralgia, vertigo, and RVM had completely resolved. She noted a decrease in previously unappreciated edema in the shoulders, forearms, back, and chest. She could lie on her side for more than 10 seconds without symptoms and could brush her hair without taking a break, both for the first time in a year. She was also able to tolerate the same physical therapy that had failed only two months prior on the right side, including stretches, muscle strengthening, and neural mobilization. After the second surgery, physical therapy was tolerated bilaterally.

Questionnaire results

**Figure 1:**
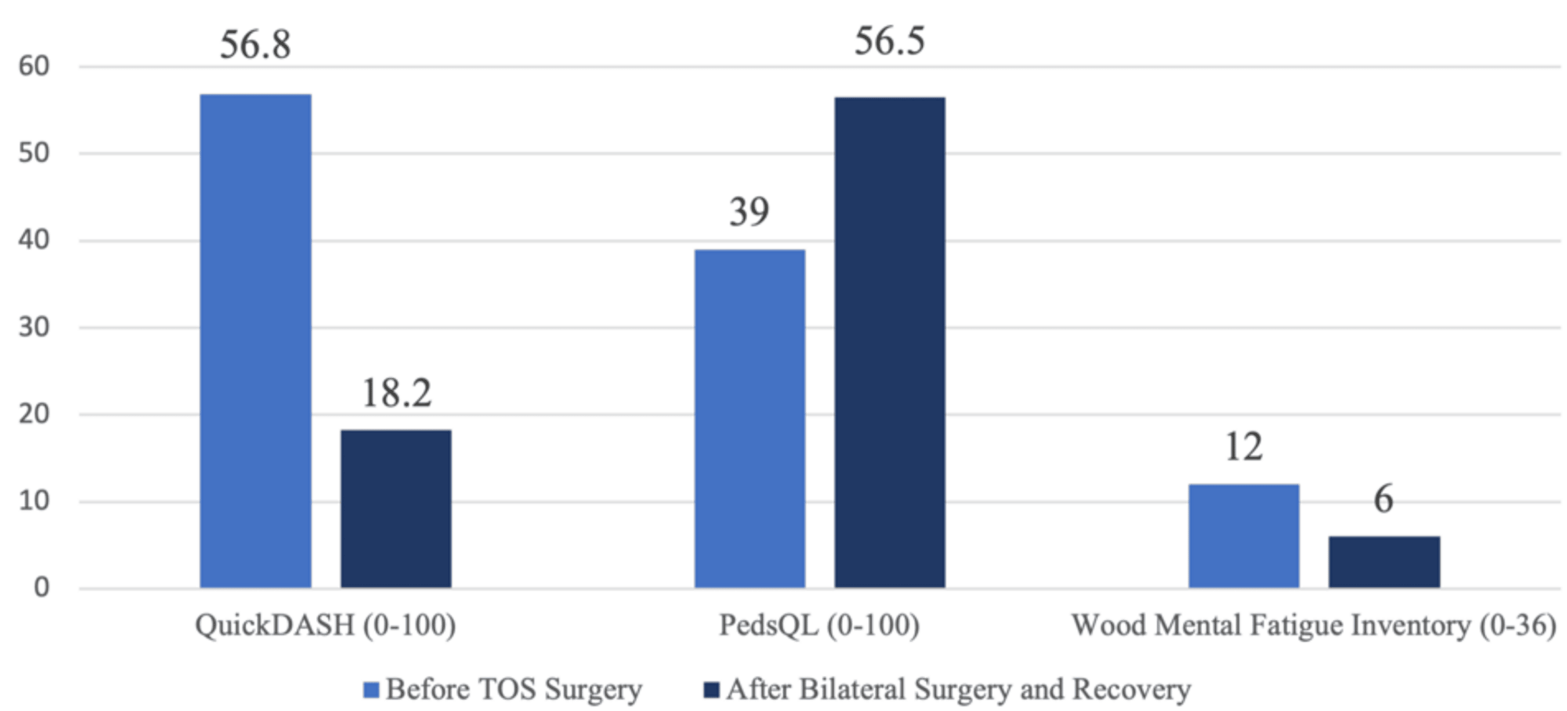
Questionnaire comparisons before and after TOS surgeries Lower scores on the QuickDASH and WMFI questionnaires, and higher scores on the PedsQL, all indicate better function TOS: thoracic outlet syndrome; WMFI: Wood Mental Fatigue Inventory; QuickDASH: Quick Disabilities of the Arm, Shoulder and Hand; PedsQL: Pediatric Quality of Life Inventory

Responses to the QuickDASH, PedsQL, and the WMFI before and after bilateral surgery and recovery are presented in Figure 1.

Physical therapy examination

Physical therapy was initiated before surgery and continued afterward, which allowed for a three-way comparison to be drawn: before either surgery and after each side was decompressed. Table 1 presents the results of provocative examination maneuvers and the range of motion compared in these three instances.

**Table 1:** Physical therapy examination comparisons before and after surgery The "+" symbol indicates an abnormal result on the test, and the "-" symbol indicates that the test is normal. For the time until symptom onset during the EAST, min stands for minutes, and s stands for seconds. All unlabeled numeric values are in units of degrees EAST: elevated arm stress test; R: right; L: left; LSLT: levator scapula length test

Physical examination maneuver	Before surgery	After left surgery	After right surgery
Adson’s test left side	+	+	-
Adson’s test right side	+	+	-
EAST left side	+ at 7 s	+ at 1 min	+ at 2 min
EAST right side	+ at 45 s	+ at 40 s	+ at 1.5 min
LSLT left side	+	-	-
LSLT right side	+	+	-
Cervical flexion	50	60	60
Cervical extension	50	70	70
Cervical rotation	R 52, L 65	R 60, L 65	R 60, L 65
Cervical side bend	R 40, L 20	R 45, L 25	R 45, L 40

Of note, the Adson’s test was positive bilaterally until both sides were decompressed. The surgery had a significant impact on the time until symptom onset during the EAST, with the left side continuing to improve even after the right-sided surgery. The LSLT and cervical movements all improved. Table 2 shows the significant improvements in grip strength after bilateral surgery.

**Table 2:** Grip strength comparison before and after TOS surgery TOS: thoracic outlet syndrome

Laterality	Grip strength before surgery (lbs)	Grip strength after bilateral surgery (lbs)
Left hand	30	64
Right hand	36.7	59.5

Table 3 illustrates the individual responses on the QuickDASH before and after bilateral surgery.

**Table 3:** QuickDASH comparison before and after TOS surgery For each question, the difficulty of the task is rated on a scale of 1-4, with 1 meaning no difficulty and 4 meaning almost impossible QuickDASH: Quick Disabilities of the Arm, Shoulder, and Hand; TOS: thoracic outlet syndrome

Question	Before surgery	After bilateral surgery and recovery
Difficulty opening a tight or new jar	5	4
Difficulty doing household chores	4	3
Difficulty carrying a shopping bag or briefcase	2	1
Difficulty washing your back	4	1
Difficulty using a knife to cut food	2	1
Difficulty with force/impact through the arm, shoulder, or hand during recreation	5	3
Extent of interference with normal social activities	4	1
Extent of limitation in work or other regular daily activities	3	1
Severity of pain in the arm, shoulder, or hand	2	1
Severity of tingling in the arm, shoulder, or hand	4	2
Difficulty sleeping due to arm, shoulder, or hand pain	1	1
Total score (0-100)	56.8	18.2

## Discussion

This report details the improvements in upper limb and systemic symptoms after bilateral surgical decompression of nTOS in a patient with hypermobile Ehlers-Danlos syndrome (hEDS) and ME/CFS. While improvements in arm fatigue, paresthesias, grip strength, and QuickDASH scores are among the expected outcomes of this surgery, there has been less emphasis in the TOS literature on the potential of surgery to lead to the kinds of improvements our patient experienced. Her other notable symptomatic changes included the resolution of occipital headaches and neuralgia, vertigo, and RVM. Moreover, there was improvement in lightheadedness during postoperative EAST and in daily life, associated with greater cognitive clarity as measured by the WMFI. All of these changes resulted in a clinically significant improvement in overall well-being and quality of life, despite the persistence of her other comorbid conditions.

Several points are worth emphasizing. First, it is now well established that headaches are common in TOS. In the Sanders series from 1964 to 1987, of 668 patients who had surgical treatment of TOS, 486 (73%) reported headaches during their initial preoperative evaluation. Of these, 94.2% were occipital, and only 5.8% were frontal alone. Later reports from Sanders and Annest [4], Sanders et al. [6], Sanders and Pearce [7], Sanders et al. [8] describe up to an 86% prevalence of occipital headaches. Raskin et al. reported a series of 30 patients with TOS, of whom 21 had headaches that appeared or became intractable at the same time as TOS symptoms. Intensification of the TOS symptoms was associated with a worsening headache, and accompanied by nausea in 18, vomiting in 11, teichopsia in 11, photophobia in nine, blurred vision in six, vertigo in five, syncope in three, and tinnitus in two [16]. More recently, Cha et al. described 50 consecutive patients with both TOS and chronic migraines (median headache duration 5.8 years), 32 of whom eventually needed TOS surgery. Twelve patients experienced complete resolution of headaches postoperatively. Of interest regarding our patient, other symptoms described by these 50 with combined TOS and migraines included visual problems in 50%, vertigo in 48%, tinnitus in 30%, and syncope or presyncope in 28% [17].

Second, an unusual feature in our patient was the occurrence of RVM, which is characterized by a rotation of 180° in the coronal plane of vision. Though this is the most common form of RVM, incomplete versions of the disorder have also been reported, such as a tilt of 90° in the sagittal plane. RVM is thought to arise from injury to the central nervous system, particularly with a high correlation to posterior circulation ischemia, but also to other causes such as vestibular dysfunction, multiple sclerosis, tumors, and seizures [18,19]. The TOS literature often describes a range of visual disturbances as a frequent symptom, but we are unaware of a description of RVM in TOS patients. We emphasize this occurrence to stimulate more complete ascertainment of this symptom in future studies of those with TOS and to ensure that TOS is included in the differential diagnosis of RVM.

Third, JH and hEDS are risk factors for ME/CFS, and there is emerging evidence that hEDS is also a risk factor for TOS. Hudson et al. described TOS symptoms in 54% of hypermobile patients presenting to a rheumatology clinic, 28% of whom had an obliteration of the radial pulse with arm abduction [20]. In our recent retrospective review of fatigued patients with symptoms suggestive of TOS, 58% had a Beighton score of four or higher, consistent with JH, and 42% had been diagnosed with hEDS [10]. Sanders et al. [6] and Sanders and Pearce [7] have also studied the histology of the TOS patients’ scalenes, finding that in all 45 cases selected, there was an increase in connective tissue fibers, an atrophy of fast-twitch muscle fibers, and an increase in slow-twitch fibers. It is unclear whether there is a relationship between these histologic abnormalities of TOS and the known histologic abnormalities of hEDS [21-25]. This gap in knowledge warrants replication of TOS scalene muscle histology studies, stratifying by the presence or absence of comorbid JH or hEDS.

Limitations

We acknowledge that the PedsQL and WMFI questionnaires were performed at the initial ME/CFS evaluation, nine months before surgery, on the day of the initial TOS diagnosis. Though these questionnaires were not repeated before surgery, the patient reported a severe increase in symptoms over that interval. We postulate that by the time of surgery, scores on these measures would have shown even greater severity in systemic symptoms.

This case report deals with a time period of approximately one year, with three months in between each surgery. Given that she is an ME/CFS patient, it is important to consider confounding treatment that could have caused the abatement of global symptoms. In this case, the only medication added was Dupixent (dupilumab) before the second surgery, which was prescribed for EoE treatment. EoE has no known effect on TOS or neurological symptoms, so we do not believe it to be a significant limitation [26].

## Conclusions

This case report emphasizes the potential for marked improvements in clinical function after recognition and surgical treatment of TOS in a patient with comorbid hEDS and ME/CFS. In addition to expected improvement in upper limb symptoms and the resolution of occipital headaches, our patient noted improvement in systemic symptoms of lightheadedness, cognitive dysfunction, and visual disturbances. This experience suggests that those with hEDS and ME/CFS should be more carefully screened for brachial plexus dysfunction. Conversely, ascertainment of systemic symptoms may enhance the diagnosis of TOS, and inform the selection of measurements in surgical outcome studies.
